# Oxygen carrier in core-shell fibers synthesized by coaxial electrospinning enhances Schwann cell survival and nerve regeneration

**DOI:** 10.7150/thno.45035

**Published:** 2020-07-11

**Authors:** Teng Ma, Yafeng Yang, Xin Quan, Lei Lu, Bing Xia, Jianbo Gao, Fengyu Qi, Shengyou Li, Laihe Zhao, Liangwei Mei, Yi Zheng, Yanbing Shen, Zhuojing Luo, Yan Jin, Jinghui Huang

**Affiliations:** 1 Institute of Orthopaedics, Xijing Hospital, Fourth Military Medical University, Xi'an 710032, China.; 2 Research and Development Center for Tissue Engineering, School of Stomatology, Fourth Military Medical University, Xi'an 710032, China.; 3 Hospital of 76th Group Army of PLA, Xining, 810000, China.; 4 Department of Orthopedics, Fourth Medical Center of Chinese PLA General Hospital, Beijing, 100048, China.; 5 Department of Plastic Surgery, Xijing Hospital, Fourth Military Medical University, Xi'an 710032, China.; 6 Department of Oral Anatomy and Physiology, State Key Laboratory of Military Stomatology, School of Stomatology, Fourth Military Medical University, Xi'an, 710032, China.; 7 Department of Orthopedics, General Hospital of Central Theater Command of PLA, Wuhan, 430070, China.

**Keywords:** Perfluorotributylamine, Coaxial electrospinning, Peripheral nerve injury, Nerve regeneration, Hypoxia

## Abstract

**Rationale:** Local hypoxia is a challenge for fabrication of cellular grafts and treatment of peripheral nerve injury. In our previous studies, we demonstrated that perfluorotributylamine (PFTBA) could provide short term oxygen supply to Schwann cells (SCs) and counteract the detrimental effects of hypoxia on SCs during the early stages of nerve injury. However, the quick release of oxygen in PFTBA compromised its ability to counteract hypoxia over an extended time, limiting its performance in peripheral nerve injury.

**Methods:** In this study, PFTBA-based oxygen carrier systems were prepared through coaxial electrospinning to prolong the time course of oxygen release. Core-shell structures were fabricated, optimized, and the oxygen kinetics of PFTBA-enriched core-shell fibers evaluated. The effect of core-shells on the survival and function of SCs was examined in both 2D and 3D systems as well as *in vivo*. The system was used to bridge large sciatic nerve defects in rats.

**Results:** PFTBA core-shell fibers provided high levels of oxygen to SCs *in vitro*, enhancing their survival, and increasing NGF, BDNF, and VEGF expression in 2D and 3D culture systems under hypoxic condition. *In vivo* analysis showed that the majority of GFP-expressing SCs in the PFTBA conduit remained viable 14 days post-implantation. We found that axons in PFTBA oxygen carrier scaffold improved axonal regeneration, remyelination, and recovery.

**Conclusion:** A synthetic oxygen carrier in core-shell fibers was fabricated by the coaxial electrospinning technique and was capable of enhancing SC survival and nerve regeneration by prolonged oxygen supply. These findings provide a new strategy for fabricating cellular scaffolds to achieve regeneration in peripheral nerve injury treatment and other aerobic tissue injuries.

## Introduction

Peripheral nerve injury (PNI) leads to loss of sensory and motor neural functions 1-3. Although autografts are considered the gold standard for the structural restoration of injured nerves, lack of available donor nerves and the impairment of donor nerve function prevent their clinical utility 4. Artificial nerve conduits offer an alternative to autografts and have been intensely studied as PNI therapeutics. Cells can be fabricated into scaffolds to promote axonal regeneration through their ability to supply key biomolecules and a hospitable microenvironment for regeneration, offering new avenues for PNI therapeutics 5, 6.

Schwann cells (SCs) are responsible for myelin-formation in the peripheral nervous system and have been fabricated onto cellular nerve grafts to improve axonal regeneration 7-9. Moreover, SCs secrete neurotrophins and produce extracellular matrix molecules to facilitate axonal outgrowth and elongation 10, 11. However, emerging evidence suggests that the functionality of transplanted SCs within nerve scaffolds is limited due to the local hypoxic environment 12, 13. Also, slow vascularization and limited oxygen diffusion into nerve grafts during transplantation limits the oxygen supply to SCs, decreasing their performance during nerve regeneration 14, 15. Therefore, enhancing oxygenation at the injury site can improve the survival and functionality of SCs within nerve grafts.

Oxygen levels can be increased within the nerve scaffolds to enhance the oxygenation of SCs prior to the ingrowth of blood vessels into scaffolds. Perfluorotributylamine (PFTBA) is a perfluorocarbon with higher oxygen solubility than water or blood 16-18. Also, as an oxygen carrier, PFTBA has biological inertness, commercial availability and can be surface sterilized to avoid contamination 19. Thus, PFTBA has drawn intense interest from researchers in regenerative medicine 20, 21. We previously demonstrated that PFTBA-enriched hydrogel could provide oxygen to cultured SCs and prevent oxygen deprivation during hypoxia 22. Thus, we identified PFTBA as a promising supplement to nerve scaffold SCs during their application for peripheral nerve injury and recovery. However, the PFTBA-hydrogel in our previous studies exhibited relatively quick release kinetics, posing limitations for sustained oxygen availability during regeneration.

Electrospinning involves applying an electrical field in the collector and spinneret through high voltages that enhance the solidification of polymers, increasing their size in the absence of post-treatment processing. In this study, we explored coaxial electrospinning for its ability to encapsulate water-soluble bioactive agents into core-shell structures, permitting the addition of bioactive agents into the fibers. We reasoned that, in hypoxic conditions, the core-shell structures could act as reservoirs for oxygen delivery using PFTBA, thereby permitting the controlled release of oxygen through the fiber shell or fiber end, enhancing the survival of SCs, and promoting nerve recovery.

**Scheme 1:**
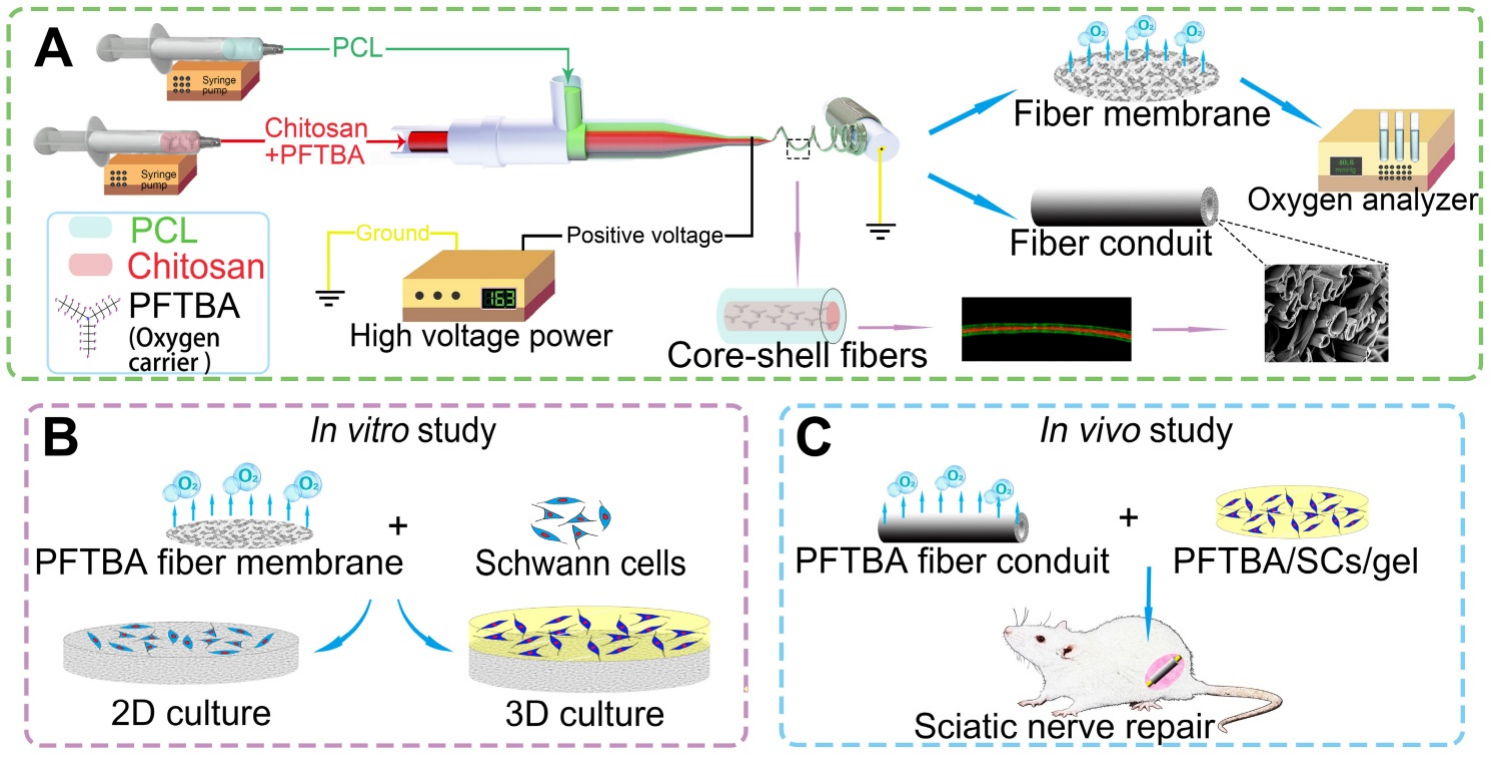
Schematic illustration of the fabrication of core-shell fibers and the preparation of conduits (A). Effects on survival and function of SCs in 2D and 3D systems (B). Application of the core-shell oxygen carrier scaffold to repair sciatic nerve defects (C).

Coaxial electrospinning, a modification of the electrospinning technique, is frequently employed to encapsulate water-soluble and fragile bioactive agents, such as DNA and growth factors, into core-shell fibers 23, 24. Core-shells have been shown to release encapsulated agents in a controlled and prolonged manner. However, it was unclear whether the core-shell structure could be a reservoir for the oxygen carrier (i.e., PFTBA), and thus permit a controlled release of oxygen via fiber shells or fiber ends during hypoxia. Such a strategy would enhance the potential of core-shell structures to promote SC survival and subsequent nerve regeneration. In this study, we fabricated the core-shell structure using coaxial electrospinning technique, and developed a coaxial electrospun fiber scaffold containing PFTBA in the core and polycaprolactone (PCL) in the shell, to permit regulated oxygen release to SCs in both 2D and 3D matrices. We also investigated the effectiveness of the scaffolds to correct 17-mm- sciatic nerve defects in rats **(Scheme 1)**.

## Methods

All Animals in this study were provided by the Experimental Animal Center of the Fourth Military Medical University. All *in vivo* studies were performed according to standard laboratory animal care guidelines (Committee for the Update of the Guide for the Care and Use of Laboratory Animals) approved by the Animal Research Committee of The Fourth Military Medical University, People's Republic of China.

### Preparation of PFTBA core and PCL shell solutions

**Figure 1:**
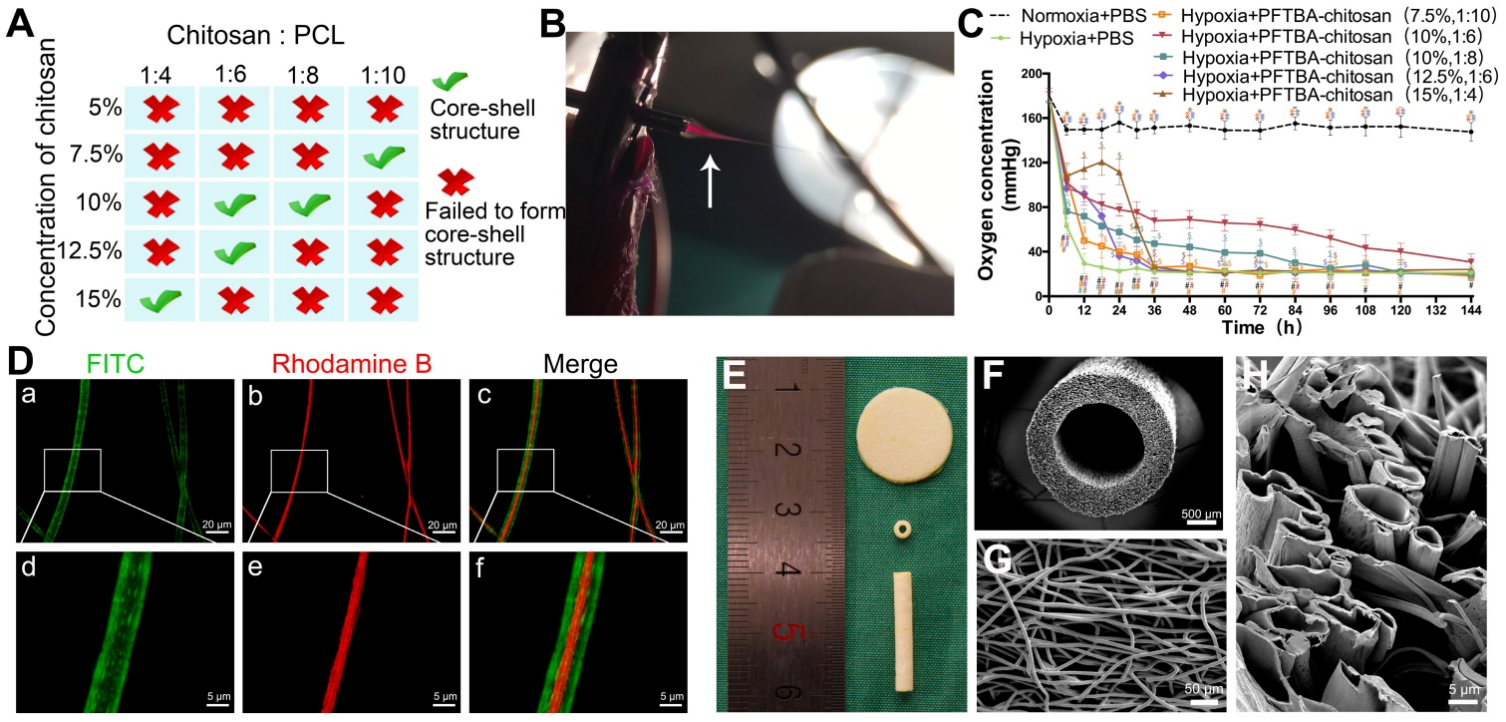
Fabrication and optimization of coaxial electrospun fibers. Parametric optimization of core-shell structures (A). Core-shell structures were fabricated by high voltage (B), see also Movie S1. Oxygen release behavior of PFTBA-encapsulated core-shell fibers (C). *p < 0.05 for comparison with Normoxia + PBS group, *^#^p < 0.05* for comparison with Hypoxia + PBS group, and^*$*^*p < 0.05* and for comparison with Hypoxia + PFTBA-chitosan (10%, 1:6) group. The microstructural appearance of core-shell fibers under fluorescence microscopy (D) and scanning electron microscopy (E-H).

The preparation of PFTBA emulsion was performed as described in previous studies with modifications 20, 22. Briefly, egg yolk lecithin (190 mg, 99% purity, Sigma) was dissolved in Tyrode's solution (1 mL, Sigma) and sonicated. PFTBA (1 mL, 98% purity, Sigma) was then added with sonication (12 times at a temperature of 4 °C). For control groups, the emulsion with PBS (without PFTBA) was prepared. To determine the optimal composition and core-shell structure, the carboxyl-chitosan (5 % w/v, 7.5 % w/v, 10 % w/v, 12.5 % w/v, and 15 % w/v.; (Sigma) **Figure 1A**) was mixed with the PFTBA emulsion to reach the core solution. Thereafter, 20 % w/v. PCL (Sigma) was prepared as the shell solution in methyl alcohol and trichloromethane 1:4 v/v (Fuyu, China). To observe core-shell fiber formation, Rhodamine B (1 mg/mL, R6626, Sigma) was mixed into the core solution and 2 mg/mL of fluorescein isothiocyanate (FITC; YESE, China) was added to the shell solution.

### Fabrication of core-shell fibers by electrospinning

We performed coaxial electrospinning using two syringe pumps (LSP02-1B, Longer, China) at high voltages. Concentric spinnerets were used for electrospinning with an outer needle of internal diameter (I.D.) 1.2 mm, and an inner needle of I.D 0.3 mm. We prepared an unventilated environment on a clean bench and moved spinnerets and the collector to it during electrospinning. It was important to avoid air turbulence to make the electrospinning process more stable. Electrospinning was monitored on a digital camera (X100f, Fujifilm, Japan, **Figure 1B**) and shell solutions were pushed via the syringe pump into the outer capillary at a rate of 0.6 mL/h. The core solution rates ranged from 0.06 to 0.15 mL/h (ratios between the core solution and shell solution: 1:10 to 1:4.). The positive voltage was fixed at 16 kV and the fibers jetted out steadily from the needle. If the positive voltage exceeded 17 kV, the strong static electric fields could cause electric shocks and device malfunction. Fibers were collected on a rotating cylinder (Ø=10 cm; 60 rpm) or steel bar (Ø=1.5mm; 180 rpm) placed 15 cm from the spinneret. The electrospun membranes and tubes (Thickness = 0.5mm) were collected and cut into appropriate sizes. The fibers with a core-shell structure were examined by fluorescence microscopy (Carl Zeiss Aixo observer A1, Germany). Conduits and membranes were imaged on an SEM (S-3400N; HITACHI, Japan) following gold coating at 25 °C and 50 % relative humidity.

### Oxygen release behavior

Each coaxial electrospun membrane with or without PFTBA was cut into circular modes of a 15.6 mm diameter and added to 24-well plates (NUNC). Because the diameters of wells were 15.6 mm, the volume of the electrospun membrane in each well was 10 pieces×0.5 mm× π (15.6 mm/2)^2^ ≈ 995.2 mm^3^ (number of membranes × thickness × area). DMEM/F12 (lacking serum, Hyclone) was added to the wells in a hypoxic incubator (0.5% O_2_/5% CO_2_) at 37 °C. Oxygen levels released from the membranes ± PFTBA were assessed on a blood-gas analyzer.

### SC culture

SCs were isolated as previously described 25. Sciatic nerves from 2-day-old Sprague-Dawley rats were treated with type 2 collagenase (1 mg/ml; Sigma) and trypsin (0.03%, Sigma) for 1 h at 37 °C. Enzyme activity was stopped through the addition of FBS, and cells were pelleted and resuspended in the mitogen medium (20 mg/ml bovine pituitary extract, 4 mM forskolin and 10 ng/ml bFGF; Sigma) and seeded onto PLL/laminin-dishes (Sigma). SCs were engineered to express GFP through retrovirus-mediated delivery 26 and cultured in the same dishes as un-transfected SCs.

### *In vitro* examination (2D)

All electrospun membranes were ^60^Co radiated at 1 kGy for sterilization. Prior to seeding, electrospun membranes were PLL/laminin coated to which 1 × 10^4^ SCs were added ± PFTBA. Cells were cultured in hypoxic or normoxic environments to permit adhesion and growth. The media was not replenished at any stage to prevent reoxygenation, and excess media was added at the initial stage. For cell viability and biocompatibility analyses, SC proliferation was assessed at days 2 and 4 by the CCK-8 assay. SC growth and apoptosis were quantitated via Annexin V-FITC/PI staining and flow cytometry analysis. The spread of SCs across the membranes was imaged by SEM. SCs were immuno-stained with the S-100 anti-body (ab52642; Abcam Inc.) to visualize cell adherence on electrospun membranes.

### *In vitro* 3D assessments

To assay the SCs in 3D cultures, a 3D matrix was generated using electrospun membranes and SCs in the fibrin gel ± PFTBA (**Figure 3B**) 20. Briefly, fibrinogen in saline (80 mg/ml) was mixed with 1×10^6^ SCs, and to achieve coagulation, the SC-fibrinogen mixture was added to thrombin (5 IU/ml)-PFTBA (10 wt. %; Sigma). SC-gel lacking PFTBA was also produced through cell-fibrinogen and thrombin-PBS mixing. SC-gel mixtures were then injected onto the membranes. The study groups included: Group 1: SCs without PFTBA injected onto the membrane in the absence of PFTBA (fibers + gel); Group 2: SCs without PFTBA injected onto the membrane with PFTBA (PFTBA fibers + gel); Group 3: SC-gel mixtures with PFTBA injected in the absence of PFTBA (PFTBA-gel); Group 4: SC-gel with PFTBA injected onto membrane with PFTBA (PFTBA fibers + PFTBA-gel).

After 48 h or 96 h of hypoxia, Live-Dead assays were performed through dual color staining (Live-Dead Cell Staining Kits, BioVision). Live-Dye^TM^ fluoresces green (ex/em 488/518 nm) in both live and dead cells, while PI stains only dead cells (ex/em 488/615 nm). Following 3D culture, SCs were counted and total RNA was extracted using Trizol (Sigma) lysis in 3D cultures. RT-PCR was then performed. Primer sequences are shown in **Table S1**.

### Nerve conduit preparation

Electrospun conduits were prepared ± PFTBA fibers, removed from the molds, and sectioned into cylinders (length: 19 mm; inner diameter: 1.5 mm; outer diameter: 2.5 mm,** Figure 1E and F**). The conduits were also sterilized with ^60^Co radiation at the intensity of 1kGy before surgery. Thereafter, SC -gel mixture (as 3D culture) was injected into the lumen of each conduit. 1) SC-gel mixture without PFTBA was injected into the conduit without PFTBA to serve as fiber conduit + SCs/gel group. 2) SCs-gel mixture without PFTBA was injected into the conduit with PFTBA to serve as a PFTBA fiber conduit + SCs/gel group. 3) SCs-gel mixture with PFTBA was injected into the conduit without PFTBA to serve as a Fiber conduit + PFTBA/SCs/gel group. 4) The SC-gel mixture with PFTBA was injected into the conduit with PFTBA to serve as a PFTBA fiber conduit + PFTBA/SCs/gel group.

### Conduit implantation

Male Sprague Dawley (SD) rats (230-250 g) were divided into 5 treatment groups (**Table S2**) and anesthetized via intraperitoneal injection of 1% sodium pentobarbital (35 mg/kg; AMRESCO, China). A skin incision of ~25 mm was introduced into the gluteal region and blunt dissection was used to expose the left sciatic nerve. Sciatic nerve segments were then removed to leave a ~17-mm-defect following nerve end retraction. In autograft groups, nerve segments were reversed 180º and re-implanted using epineurial monofilament sutures. SC-gels (not GFP-SCs) were used to treat the defects in the other four groups. Stumps (proximal and distal) were inserted ~1 mm into the lumen and sutured to the conduit wall. The skin was closed with single stitches and rats were replaced into cages. Rats were allowed to recover with food and water ad libitum.

### Post-surgical cell viability assessments

At 7 and 14 days post-implantation, animals were anesthetized and intracardially perfused for 1 min with 0.1 M phosphate buffer and ice-cold 4% paraformaldehyde (PFA, Aladdin, China) in 0.1 M phosphate buffer for 30 min. Nerve segments were dissected and fixed for another 4 h in the same fixative solution. After cryoprotection in 30% sucrose at 4°C, sections were cut on a vibratome VT1000A (25 μm-thick sections) and mounted **(Figure 4F)**. GFP-SCs were examined via confocal microscopy (BX-51; Olympus, Japan) and five transverse sections from the mid-portion of scaffold quantified. The average number of viable cells per section were then counted.

### Behavioral assays

Recovery of function in the hindlimb was assessed using the Sciatic Function Index (SFI) at 6 and 12 weeks post-treatment 15. Briefly, hind-paws were inked and rats allowed to walk down a 90-cm-long track. SFI values were then calculated as follows:

SFI = [-38.3×(EPL-NPL)/NPL]+[109.5×(ETS-NTS)/NTS]+[13.3×(EIT-NIT)/NIT]-8.8

Print length (PL): distance from the heel to the top of the third toe; intermediary toe spread (IT): 2^nd^ to 4^th^ toe distance; toe spread (TS): first to fifth toe difference. N: non-operated foot; E: operated, experimental foot. SFI values of 0 were suggestive of improved recovery rates. Values close to -100 were deemed as completely dysfunctional.

Following track analysis, plantar tests were performed to assess heat hypersensitivity and sensory function recovery in the injured hindlimb. For the assessments, animals were placed in clear Plexiglas boxes for ≥1 h for habituation prior to assessments. Radiant heat was then measured in the left hind paws and withdrawal times (s) were recorded in response to heat. Animals withdrawing their paws within 30 s received no further stimulation to prevent thermal injury. Experiments were repeated four times with intervals of 7 min between each application.

### Electrophysiological examination

Postoperatively at 6 and 12 weeks, sciatic nerves were stimulated following anesthesia with a monopolar needle electrode (cathode) and cup electrode (anode). Cathodes were used to stimulate ~2 cm areas of shaved skin and recordings were made from the mid gastrocnemius to the tendon using active reference electrodes. Compound muscle action potentials (CMAPs) were measured on a PowerLab 4SP distal data acquisition system (Keypoint 3.02). For quantitation, CMAPs, nerve conduction velocities (NCV) and the latency of CMAP onset were assessed.

### Fluoro-Gold (FG) retrograde tracing

Neurons that regenerated across the conduit into the distal nerve stump were measured through retrograde labeling 27. After 6 and 12 weeks of surgery, 4% FG solution (2 μl) was injected into the rat sciatic nerve trunk at a location ~5 mm distal to the grafts and the wounds were closed. After 1 week of retrograde labeling, rats were euthanized and intracardially perfused with 4% (w/v) PFA. Lumbar enlargement of the regions possessing the sciatic nerve motor neurons was observed and L4, L5 and L6 DRGs were harvested and fixed in 4% PFA plus 30% sucrose for 5 days. Samples were sectioned on a cryostat to 50 μm and 20 μm for spinal cord and DRG regions. Samples were mounted onto glass slides and imaged (BX-51; Olympus, Japan). FG-labeled DRG sensory neurons and FG-labeled spinal cord motor neurons were then counted.

### Morphometric analysis of axonal regeneration

Regenerated nerves in each group were macroscopically imaged with a camera (X100f, Fujifilm) after 0, 6, and 12 weeks of regeneration. Regenerated nerves were collected and fixed in 2% glutaraldehyde, followed by post-fixing in 1% osmium tetroxide. Nerves were subjected to a gradient ethanol series and epoxy resin embedded. Sections (~1.0 μm or ~50.0 nm for ultrathin sections) were stained with 1% methylene blue and imaged. Ultrathin sections were stained using lead citrate and uranyl acetate and assessed by TEM (Thermo, TECNAI Spirit). For quantifications, a total of 5 sections (~1.0 μm or ~50.0 nm thickness) were randomly selected from the regenerated nerves and regeneration was assessed using the following parameters: 1) distal to proximal nerve area, 2) the number of myelinated axons/mm^2^ area, and 3) mean nerve fiber diameter per section. Myelination was calculated from the mean axon-to-fiber diameter (G-ratio). Morphological assessments were performed by a blinded investigator.

### Immunohistochemistry (IHC)

The primary SCs separated from 2-day-old SD rats and cultured for three passages were injected into the lumen of conduits. Twelve-week post-surgery, immunohistochemistry using S100 (ab52642; Abcam Inc., UK) and NF160 (2838S; CST, USA) antibodies was performed to assess the distribution of migrated SCs and regenerated axons in the conduit. Briefly, nerves were fixed in 4% PFA with 30% sucrose, and sectioned. Sections were treated with 0.2% Triton X-100 for 10 min and blocked in 0.1% BSA for 30 min. Sections were then probed with primary antibodies against S100 and NF160 overnight at 4 °C. Sections were washed and labeled with IgG TRITC secondary antibodies (ab150080; Abcam Inc., UK) and IgG FITC (ab150113; Abcam Inc., UK) for 2 h. Samples were mounted onto glycerin-coated slides and imaged (**Figure 6A**).

### Muscle histology

Operated gastrocnemius muscles from the hind limbs were harvested 12 weeks after the surgery and fixed in PFA. A total of 5 sections in the traverse plane (~50.0 μm) for each sample were Masson stained and imaged. A total of 5 middle-powered fields (×200 magnification) per section were selected and analyzed on Image-Pro Plus 6.0. Atrophy and re-innervation were quantified by the % of muscle fiber area (P_m_), calculated as:

P_m_ = A_m_/A_t_ × 100%

A_m:_ muscle fiber area in each field; A_t:_ total area of muscle fibers and tissues.

### Statistical analysis

Data were analyzed using SPSS 13.0 and as the mean ± SD. Data were compared through a one-way ANOVA with Bonferroni tests for pairwise comparisons of treatments and times. *P-values<0.05* were deemed significant differences.

## Results

### Preparation and optimization of coaxial electrospun fibers

The coaxial electrospun fibers were fabricated according to the parameters presented in **Figure 1A**. During the electrospinning, we found that the low voltage (≤12 kV) was unable to push the core and shell solutions to form a stable Taylor cone, which is important in coaxial electrospinning. After increasing the voltage from 12 kV to 16 kV, the fibers jetted steadily out from the needle. However, if positive voltage exceeded 17 kV, it was hazardous for the equipment and unsafe for experimenters as the strong static electric fields can cause electric shocks and device malfunction. Thus, we chose 16 kV as a suitable voltage in our study. After fixing the positive voltage at 16 kV, videos on the tip of the coaxial needle were recorded to observe core-shell fiber formation and optimize the parameters of coaxial electrospinning. As shown in **Scheme 1A, Figure 1B,** and **Movie S1**, the Taylor cone could be observed and the fiber formation process was stable (chitosan: PCL, 1:6). With the increasing ratio of chitosan in the core (chitosan: PCL, 1:4 and 1:2) , the core solution cut off the supply of shell solution intermittently, resulting in unstable core-shell structures during the coaxial electrospinning process (**Movie S2 and S3, Figure S1A-C and D-F**). When the ratio of chitosan to PCL decreased to 1:10 (**Movie S4, Figure S1G-I**), the shell solution was electrospun into PCL fibers only, resulting in the clustering of fibers and loss of core-shell structure. When the fibers with core-shell structure were examined by fluorescence microscopy **(Figure 1D)**, only 5 sets of parameters could form the core-shell structure by the coaxial electrospinning technique **(Figure 1A)**.

The ability to release oxygen by core-shell fibers, which were fabricated using different chitosan concentrations and chitosan/PCL ratios, was assessed at the indicated times over 6 days. **Figure 1C** shows that the oxygen level in membranes lacking PFTBA rapidly declined after 12 h of hypoxia. In contrast, following the addition of PFTBA to the core, higher oxygen levels were observed for 36 h compared to membranes lacking PFTBA. Furthermore, at 36 h after hypoxic exposure, the oxygen level in the core-shell fibers (10% chitosan; chitosan: PCL, 1:6) with PFTBA was higher than other core-shell fibers from 36 h to 144 h. Following 6 days of hypoxia, PFTBA-encapsulated core-shell fibers showed exhausted oxygen levels in a range comparable to the core-shell fibers. Therefore, 10% chitosan and 1:6 chitosan: PCL parameters were selected for subsequent experiments as high oxygen levels could be obtained during the sustained oxygen release by PFTBA in the core-shell structure **(Figure 1E).** The fiber conduit appeared as a hollow tube of ~≥19.0 mm long with an outer diameter of 2.5 mm and an inner diameter of 1.5 mm **(Figure 1E and F)**. Core-shell structures were also found on the coaxial electrospun fiber cutting surface of the conduit** (Figure 1G and H)**. The outer and inner diameters of core-shell were 7.54 ± 2.15 μm and 3.16 ± 2.43 μm, respectively **(Figure 1D and H)**.

### Influence of core-shell fibers on SC survival and proliferation under hypoxic conditions

**Figure 2:**
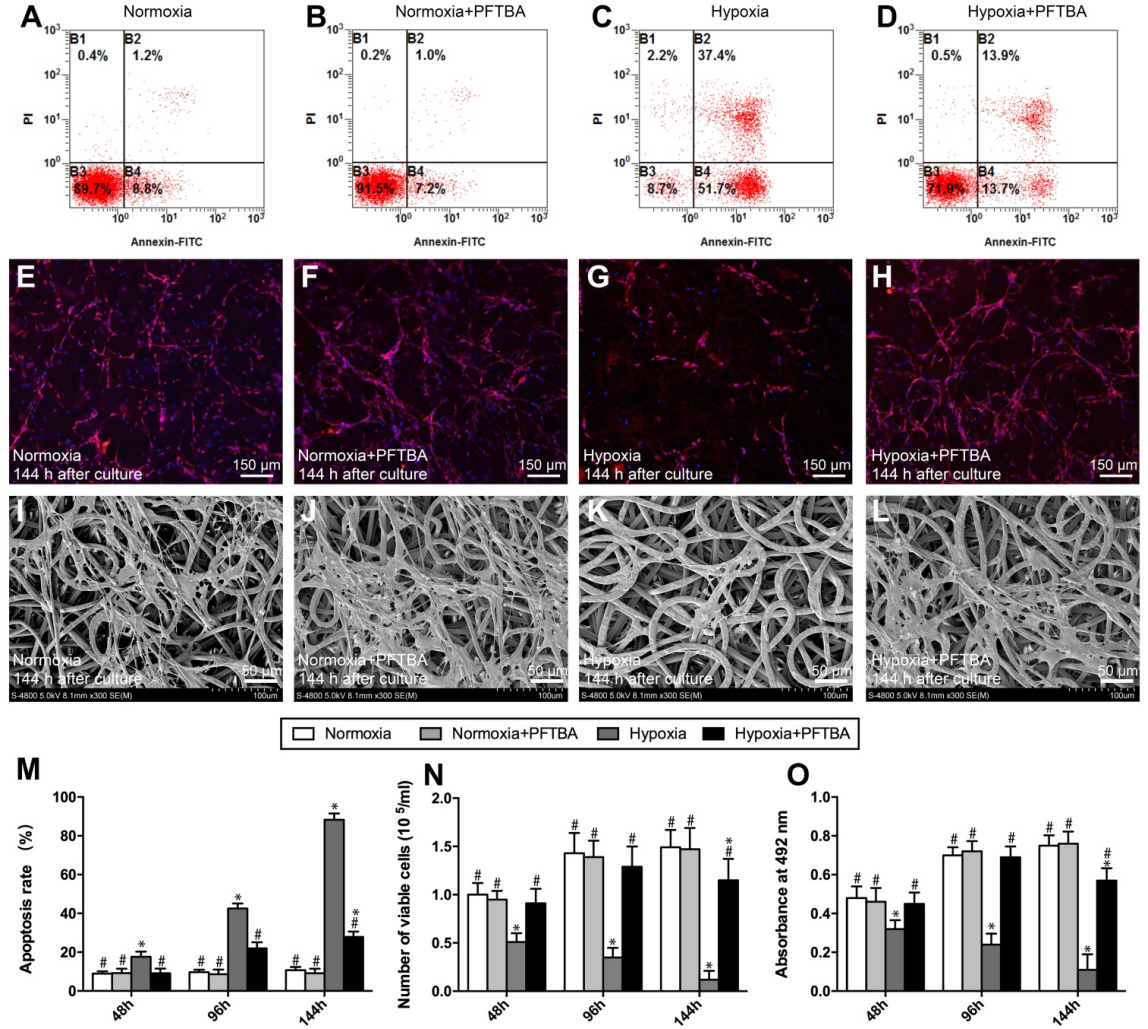
Representative images of apoptosis assay (A-D), DAPI staining (E-H) and SEM photomicrographs (I-L) in each group. Apoptosis assay (M), Schwann cell number count (N), and CCK-8 values (O) in each group were obtained by averaging the results of four samples from each group. All data are expressed as mean ± SD (J). *p < 0.05 for comparison with the Normoxia + PFTBA group.^*#*^*p < 0.05* for comparison with the Hypoxia group.

We previously demonstrated that PFTBA-enriched hydrogels could provide oxygen to cultured SCs to prevent oxygen deprivation during hypoxia. However, a limitation of the PFTBA-hydrogels was their quick release kinetics, which posed limitations in providing sustained oxygen in the early stage of regeneration. In this study, to examine the influence of released oxygen on cell survival, SCs were cultured on the electrospun core-shell fibers. In a normoxic environment, the numbers of apoptotic cells were comparable with or without PFTBA **(Figure 2A, B and M)**. Upon clustering under hypoxic conditions, the percentage of apoptotic cells lacking PFTBA was significantly higher than that with PFTBA **(Figure 2C, D, and M)**.

The numbers of surviving SCs in each group were counted at 48 h, 96 h, and 144 h after oxygen deprivation. Under the normoxic environment, SC survival was comparable across the groups ± PFTBA (*p>0.05*, **Figure 2E, F and N**). In hypoxic conditions, however, SC numbers were higher in PFTBA vs non- PFTBA groups (*p<0.05*, **Figure 2G, H, and N**). SC proliferation was then assessed by the CCK-8 assay. Normoxia groups with PFTBA were comparable to non-PFTBA groups (*p>0.05*, **Figure 2O**. Under hypoxic conditions, CCK-8 values in the PFTBA group exceeded those of the non-PFTBA group (*p<0.05*, **Figure 2O**). At 144 h after culture, the morphological characteristics of SCs on the core-shell fibers were examined by SEM** (Figure 2I-L)**. SCs on core-shell fibers without PFTBA shrunk with damaged membranes and surface particulates **(Figure 2K)**. In contrast, SCs attached and spread well on the core-shell fibers with PFTBA, confirming the results observed in the apoptosis assay **(Figure 2J and L)**.

### Effects of PFTBA core-shell fibers on SC functionality in 3D cultures in a hypoxic environment

**Figure 3:**
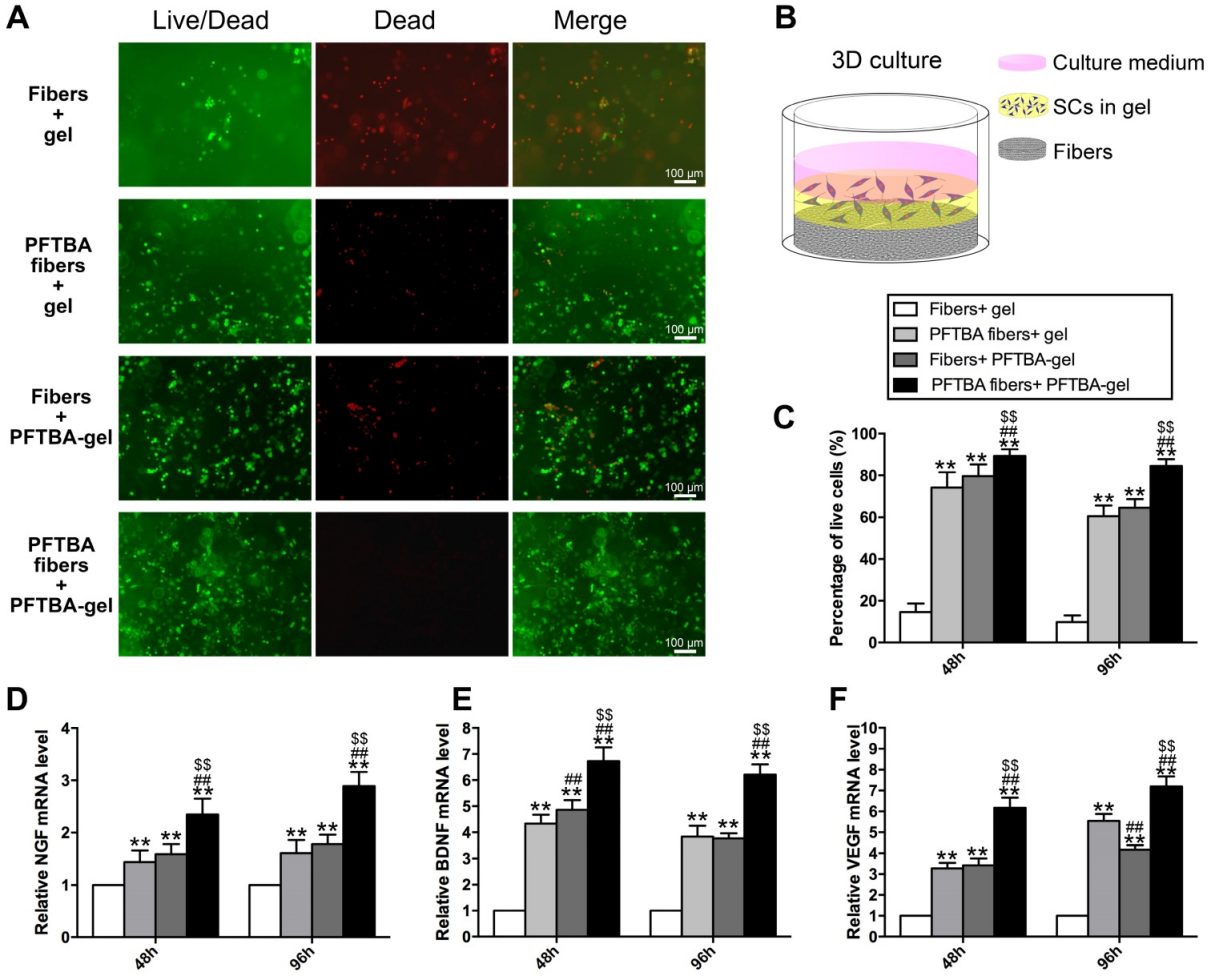
Representative images of Live-Dead staining of the 3D cultured Schwann cells in each group (A). Schematic diagram of the process of preparing the 3D culture matrix (B). Percentages of living SCs for each group are shown in (C). mRNA levels of NGF (D), BDNF (E), and VEGF (F) in each group at 48 and 96 h after hypoxia.

We further assessed the influence of PFTBA-containing core-shell fibers on SC survival in 3D *in vitro* models using the Live-Dead staining assay **(Figure 3A)**. The PFTBA-enriched gel was introduced into 3D culture medium **(Figure 3B)** to compare with our previous study 22. The numbers of live cells were highest in the PFTBA fibers + PFTBA gel group, followed by the PFTBA fibers + gel and fiber conduit + PFTBA gel group, and was lowest in the fibers + gel group (*p<0.05*, Figure 3C). These data highlighted the synergistic effects of PFTBA-enriched gel and PFTBA-encapsulated fibers on enhancing SC survival in 3D cultures under hypoxia.

We next examined the expression of NGF, BDNF and VEGF and detected higher expression at the mRNA levels in the PFTBA fibers + PFTBA gel group than in PFTBA fibers + gel, fibers + PFTBA gel, and fibers + gel groups (*p<0.05*, **Figure 3D-F**). The expression of NGF, BDNF, and VEGF in the PFTBA fibers + gel and fibers + PFTBA gel groups was also higher than in the fibers + gel group (*p<0.05*, **Figure 3D-F**).

### PFTBA oxygen carrier system enhances survival of SCs *in vivo*

In this study, we encapsulated PFTBA in the core-shell fibers to prolong the time course of oxygen release and also injected PFTBA-gel to get more oxygen carrying capacity. PFTBA in core-shell fibers and gel synergistically enhanced the survival of SCs following *in vivo* implantation. Three-dimensional (3D) cell cultures have been shown to better mimic physiological conditions in the body. To assay the SCs in the 3D culture system, the gel was injected onto the membrane with or without PFTBA. We found that cell survival was highest in the PFTBA fibers + PFTBA gel group. The PFTBA-gel in our previous studies showed relatively quick release kinetics posing limitations in providing sustained oxygen during regeneration 22. To prolong the time course of oxygen release in this study, we injected PFTBA gel, together with the encapsulated PFTBA in core-shell fibers, to obtain more oxygen carrying capacity. We observed a synergistic enhancement of SC survival following *in vivo* implantation of PFTBA in core shell fibers and gel. We, therefore, subsequently added the PFTBA-gel in this conduit to increase SC survival *in vivo*.

**Figure 4:**
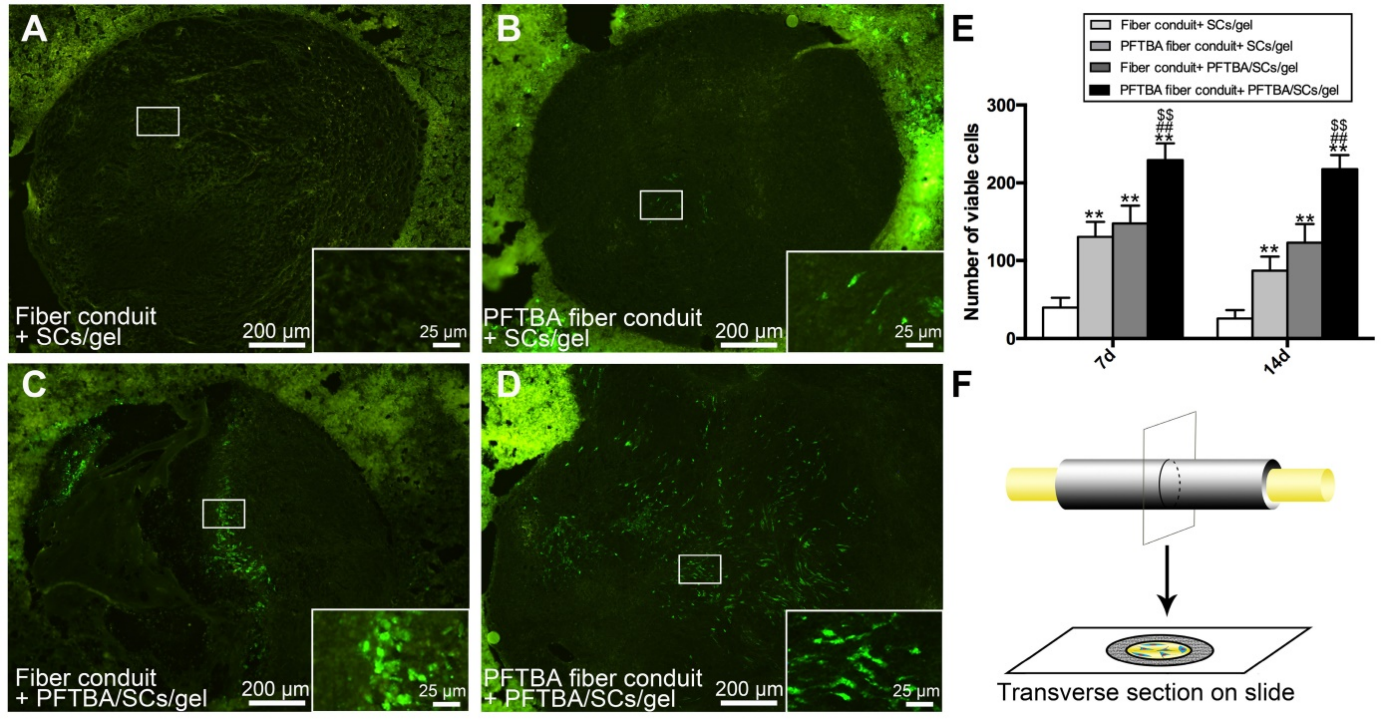
Number of GFP- SCs at 14 days after surgery in fiber conduit + SCs/gel (A), PFTBA fiber conduit + SCs/gel (B), Fiber conduit + PFTBA/SCs/gel (C), and PFTBA fiber conduit + PFTBA/SCs/gel (D) groups. Schematic of the process of preparing the scaffold sections (F). All data are expressed as mean ± SD (E). *p < 0.05 and **p < 0.01 for comparison with Conduit + gel group. *^#^p < 0.05* and *^##^p < 0.01* for comparison with PFTBA conduit + gel group. *^$^p < 0.05* and^*$$*^*p < 0.01* for comparison with Conduit + PFTBA gel group.

To study the effects of the newly designed oxygen carriers on the survival of SCs *in vivo,* GFP-labeled SCs were counted at 7 and 14 days after conduit implantation. As displayed in **Figure 4A-D and F**, after 7 and 14 days of implantation, SC numbers were highest in PFTBA fiber conduit + PFTBA gel **(Figure 4D)**, followed by PFTBA fiber conduit + gel **(Figure 4B)** and fiber conduit + PFTBA gel **(Figure 4C)**. SC numbers were lowest in the fiber conduit + gel group **(Figure 4A)**. The results indicated that PFTBA in core-shell fibers and hydrogel synergistically enhanced the survival of SCs following *in vivo* implantation.

### PFTBA oxygen carrier system promotes axonal regeneration

**Figure 5:**
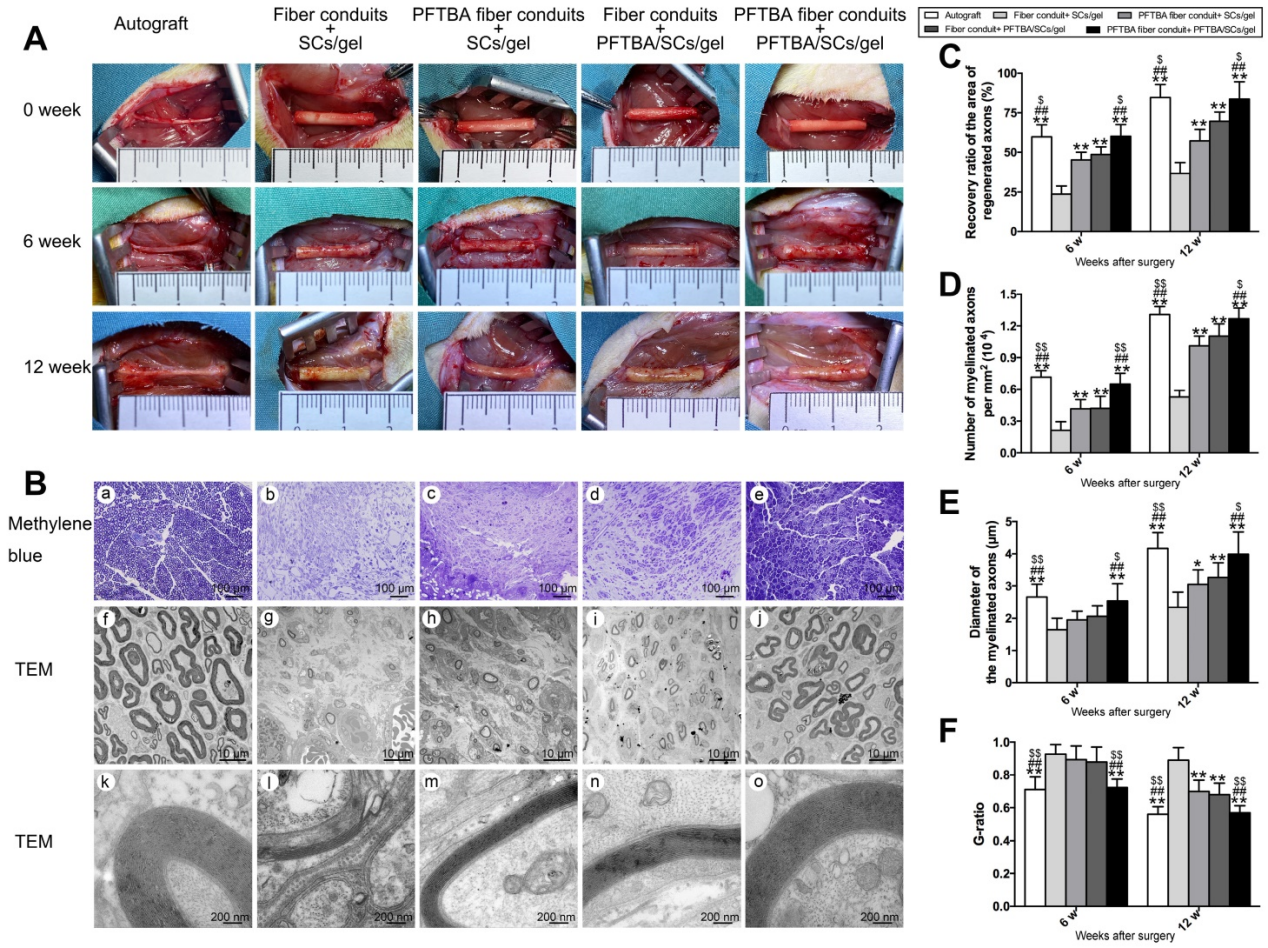
Morphology of regenerated nerves. Observation on macroscopic level of regenerated nerve tissues (A). Representative regenerated axons in the distal conduit at 12 weeks postoperatively (B). Representative transmission electron micrographs of regenerated axons (Bf-j), and myelin sheath (Bk-o) in the distal conduit in each group. % of distal/proximal nerve area (C), number of myelinated axons (D), diameter (E), and G-ratios (F) in the distal portion of the conduit. *p < 0.05 and **p < 0.01 for comparison with Fiber conduit + SCs/gel group, *^#^p < 0.05* and *^##^p < 0.01* for comparison with PFTBA fiber conduit + SCs/gel group, and *^$^p < 0.05* and *^$$^p < 0.01* for comparison with Fiber conduit + PFTBA/SCs/gel group.

During PNI, the blood circulation is disrupted and ischemia occurs at the injury site, leading to a transient hypoxic environment prior to revascularization. The exposure of nerve scaffolds to hypoxic conditions results in nerve and seed cell necrosis, decreased functionality, and a subsequent loss of nerve regeneration. We observed the gross morphology of regenerated axons (Figure 5), and the nerves in each group were macroscopically imaged under a camera after 0, 6, and 12 weeks of regeneration (Figure 5A). A large number of myelinated and unmyelinated axons were observed at each time point in PFTBA fiber conduit + PFTBA/SCs/gel group **(Figure 5Be, j and o)** compared to PFTBA fiber conduit + SCs/gel **(Figure 5Bc, h and m)**, conduit + PFTBA/SCs/gel **(Figure 5Bd, i and n)**, and fiber conduit + SCs/gel groups **(Figure 5Bb, g, l)**. The recovery area of the regenerated axons **(Figure 5C)**, myelinated axons per mm^2^** (Figure 5D)**, and the myelinated axon diameter (**Figure 5E**) were found to be highest in the PFTBA fiber conduit + PFTBA/SCs/gel group, followed by the PFTBA fiber conduit + SCs/gel and conduit + PFTBA/SCs/gel groups, and least in the fiber conduit + SCs/gel group. The mean G-ratio in the PFTBA fiber conduit + PFTBA/SCs/gel group was less than the PFTBA fiber conduit + SCs/gel, conduit + PFTBA/SCs/gel and fiber conduit + SCs/gel groups (*p<0.05*, **Figure 5F**), suggesting greater levels of myelination in the PFTBA fiber conduit + PFTBA/SCs/gel group. These data indicated that PFTBA in core-shell fibers and hydrogel synergistically enhanced axonal regeneration and remyelination in the repair of peripheral nerve defects.

**Figure 6:**
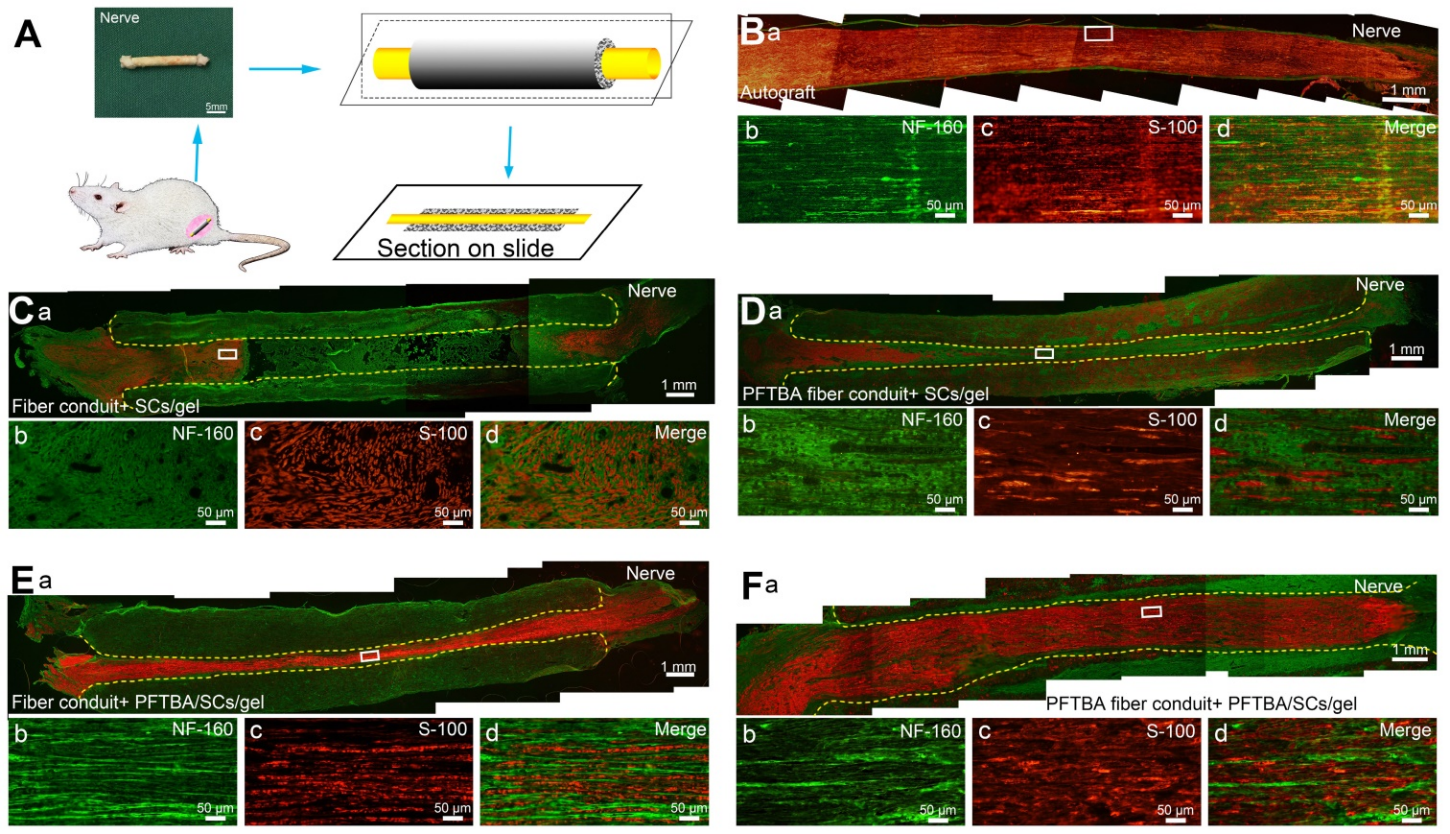
Double S-100/Neurofilament-160 analysis of regenerated nerves. Schematic of the process of preparing the conduit sections (A). Sections of conduits in the Autograft group (Ba-d), Fiber conduit + SCs/gel group (Ca-d), PFTBA fiber conduit + SCs/gel group (Da-d), Fiber conduit + PFTBA/SCs/gel group (Ea-d), and PFTBA fibers conduit + PFTBA/SCs/gel group (Fa-d) at 6 weeks postoperatively. Dashed lines distinguish regenerated nerves from conduits.

IHC assays showed that migrated SCs and regenerated axons distributed evenly in the autografts and PFTBA fiber conduit + PFTBA/SCs/gel after 6 weeks of surgery **(Figure 6B and F)**. An improved morphological appearance was also observed in the PFTBA fiber conduit + SCs/gel **(Figure 6Db-d)** and conduit + PFTBA/SCs/gel groups **(Figure 6Eb-d)** than in the control fiber conduit and gel group **(Figure 6Cb-d)**, confirming their ability to enhance axonal regeneration *in vivo*.

### PFTBA oxygen carrier system promotes neurologic functional recovery

**Figure 7:**
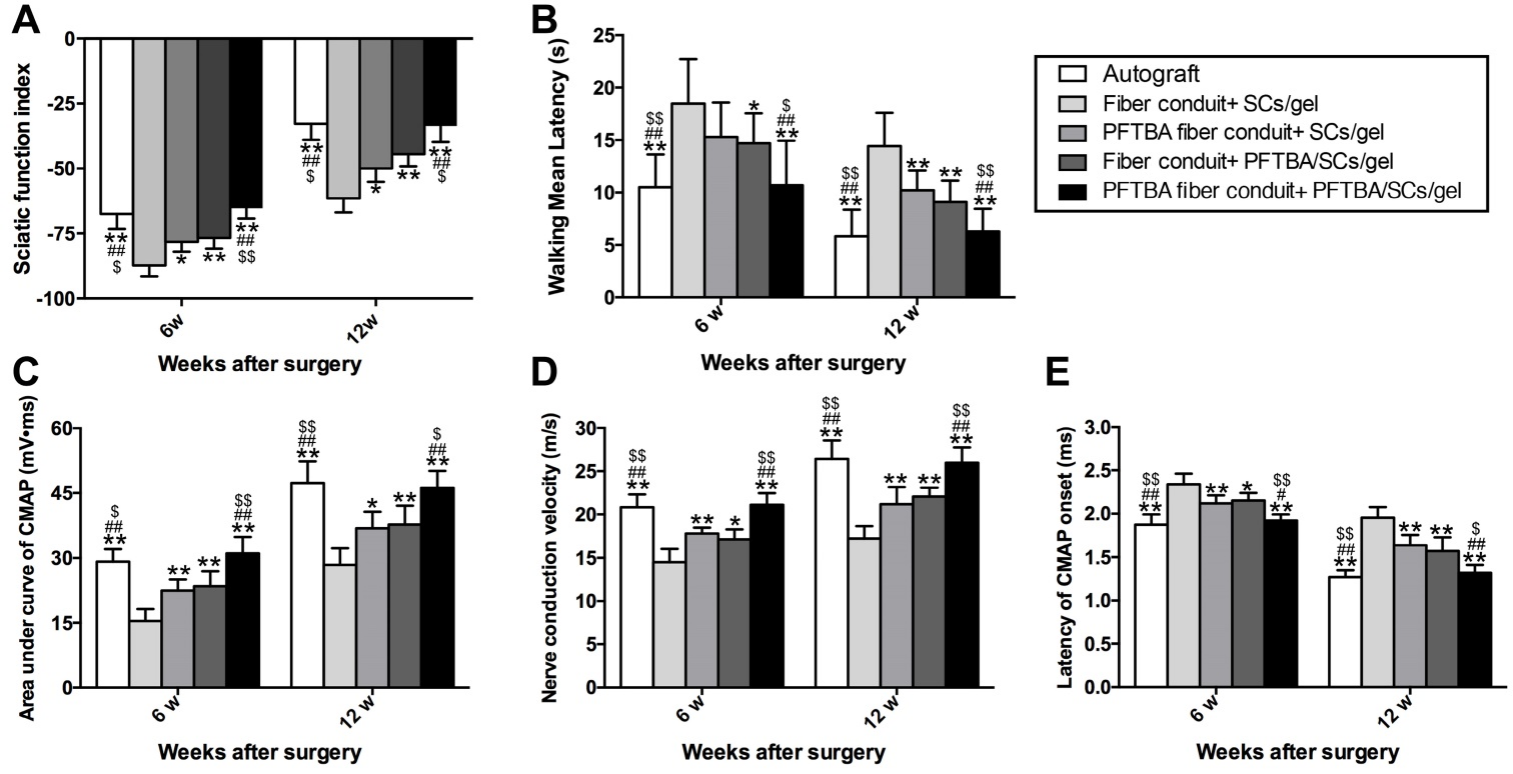
Functional index (A) and walking mean latency (B) in each group. Neuro-electrophysiologic assessments in each group (C-E). The area under the curve of CMAP (C), NCV value (D), and latency of CMAP onset (E) recorded at 6 and 12 weeks after surgery. All data were expressed as mean ± SD. *p < 0.05 and **p < 0.01 for comparison with Fiber conduit + SCs/gel group, *^#^p < 0.05* and *^##^p < 0.01* for comparison with PFTBA fiber conduit + SCs/gel group, and *^$^p < 0.05* and *^$$^p < 0.01* for comparison with Fiber conduit + PFTBA/SCs/gel group.

From behavioral and electrophysiological assessments, the SFI was highest in the PFTBA fiber conduit + PFTBA/SCs/gel, followed by PFTBA fiber conduit + SCs/gel, conduit + PFTBA/SCs/gel, and lowest in the conduit + SCs/gel group (*p<0.05,* **Figure 7A**). The PFTBA fiber conduit +PFTBA gel also achieved significantly higher CMAPs, higher NCVs, and shorter CMAP latency than PFTBA fiber conduit + SCs/gel, conduit + PFTBA/SCs/gel, and conduit + gel groups at 6 and 12 weeks post-surgery (*p<0.05,* **Figure 7C-E**). These data suggested that PFTBA fiber conduit with inner PFTBA/SCs/gel improved functional recovery following nerve defects in rat models.

Plantar assessments were used to monitor sensory functional recovery 6- and 12- week post-surgery. The PFTBA fiber conduit + PFTBA/SCs/gel group exhibited higher levels of functional recovery, with more rapid responses to thermal stimuli compared to PFTBA fiber conduit + SCs/gel, fiber conduit + PFTBA/SCs/gel, and fiber conduit + SCs/gel (*p<0.05*, **Figure 7B**). The walking mean latencies were similarly comparable across the fiber conduit + PFTBA/SCs/gel and fiber conduit + SCs/gel (*p>0.05*, **Figure 7B**), but shorter than the fiber conduit + SCs/gel (*p<0.05*, **Figure 7B**). These data collectively suggested that PFTBA fiber conduits with inner PFTBA/SCs/gel promoted functional sensory recovery in *in vivo* rat models.

### Nerve fiber regeneration assay

**Figure 8:**
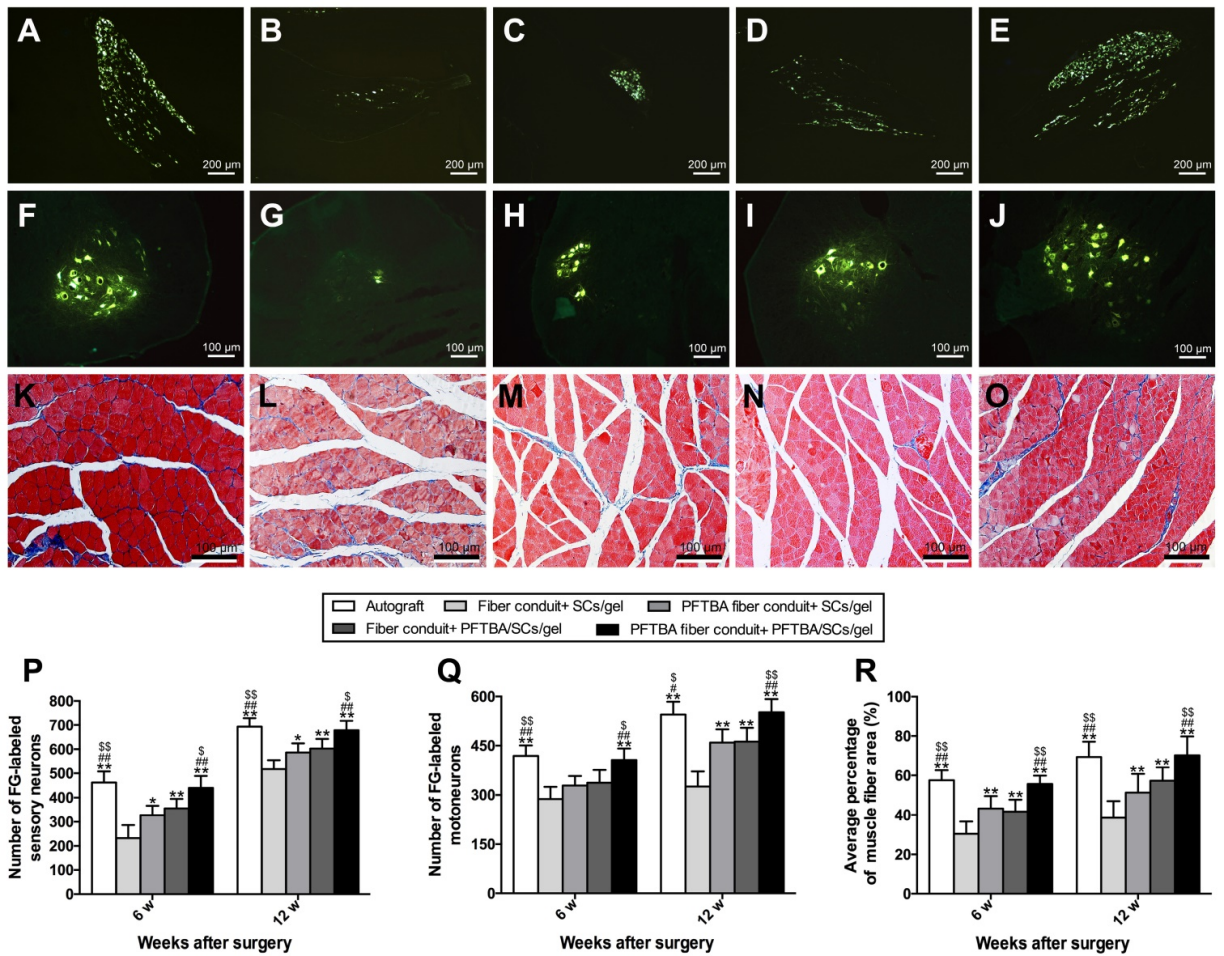
Fluorescent gold-labeled sensory neurons in DRG (A-E), motoneurons in spinal cord (F-J), and transverse-sectioned gastrocnemius muscle following Masson staining in the Autograft group (A, F and K), Fiber conduit + SCs/gel group (B, G and L), PFTBA fiber conduit + SCs/gel group (C, H and M), Fiber conduit + PFTBA/SCs/gel group (D, I and N) and PFTBA fiber conduit + PFTBA/SCs/gel group (E, J and O) at 12 weeks postoperatively. The average number of fluorescence-positive sensory and motoneurons neurons in each group are shown in (P) and (Q), respectively. The average percentage of muscle fibers in each group are shown in (R). All data expressed as mean ± SD. *p < 0.05 and **p < 0.01 for comparison with Fiber conduit + SCs/gel group, *^#^p < 0.05* and *^##^p < 0.01* for comparison with PFTBA fiber conduit + SCs/gel group, and^*$*^*p < 0.05* and *^$$^p < 0.01* for comparison with Fibers conduit + PFTBA/SCs/gel group.

We identified the numbers of regenerated neurons by FG retrograde labeling. Dorsal root ganglion and spinal cord anterior horn sections were positive for FG fluorescence at the indicated time points. The numbers of FG-labeled motor and sensory neurons peaked in the PFTBA fiber conduit + PFTBA/SCs/gel and autograft groups, followed by the PFTBA fiber conduit + SCs/gel and conduit + SCs/gel groups, and was lowest in the fiber conduit + SCs/gel group (*p<0.05*, **Figure 8A-J, P and Q**). This indicated high levels of neuronal regeneration into the distal stumps of PFTBA fiber conduit + PFTBA/SCs/gels and autograft groups.

**Figure 8K-O** shows the percent of muscle fiber areas that were comparable across PFTBA fiber conduit + PFTBA/SCs/gel and autograft groups (*p>0.05*, **Figure 8R**), but higher than PFTBA fiber conduit + SCs/gel, conduit + PFTBA/SCs/gel and conduit + SCs/gel groups (*p<0.05*, **Figure 8R**) at 12 weeks post-surgery. The % of muscle fiber area was also comparable across the fiber conduit + PFTBA/SCs/gel and fiber conduit + SCs/gel (*p>0.05*, **Figure 8R**), but higher than fiber conduit + SCs/gel groups (*p<0.05*, **Figure 8R**). Taken together, these data suggested that PFTBA fiber conduit + PFTBA/SCs/gel can decrease muscle atrophy following nerve repair.

## Discussion

Peripheral nerve injury leads to loss of sensory and motor neural functions. For PNI treatments, artificial nerve conduits offer an alternative to autografts and have been intensely studied. Supportive cells can be introduced into scaffolds to promote axonal regeneration through their ability to supply key biomolecules and a favorable microenvironment for regeneration, offering new avenues for PNI therapeutics. In the present study, we first fabricated PFTBA-encapsulated core-shell fibers using the coaxial electrospinning technique. PFTBA core-shell fibers were used to construct a PFTBA-based oxygen-carrying system and its effect on survival of SCs *in vitro* and regeneration of axons *in vivo* was examined. We found that PFTBA-encapsulated fibers could release oxygen for ~144 h, improving SC survival under hypoxic conditions assessed in 2D and 3D culture systems. Furthermore, *in vivo* studies showed that PFTBA-based oxygen carriers improved nerve recovery in rat models of nerve defects. These findings indicated that the PFTBA-based oxygen carriers could circumvent the hypoxic environment by enhancing oxygen transfer, improving the survival and functionality of implanted SCs, and underscoring the possibility of using the PFTBA-based, oxygen-carrying system for protecting SCs and accelerating axonal recovery during PNI treatment.

It has been shown that the blood circulation is disrupted and ischemia occurs at PNI sites, leading to a transient hypoxic environment prior to revascularization 15, 28, 29. Also, hypoxia ensues as a consequence of defects in blood supply at the peripheral nerve injury site, leading to degradation and necrosis of the surrounding tissues. Therefore, after trauma, hypoxia is not only restricted to peripheral nerves, but is a common issue for different tissues. The exposure of nerve scaffolds to hypoxic conditions results in nerve and seed cell necrosis, decreased functionality, and a subsequent loss of nerve regeneration 30-32. PFCs have been intensely investigated to address these issues due to their high solubility for oxygen and ability to provide oxygen supply to hypoxic and damaged tissues. PFCs in a liquid-liquid phase system can transport oxygen to cells, improving cell growth and viability in various cell types, including plant and bacterial cell cultures 19. Furthermore, PFC emulsions can overcome hypoxia-induced damage to cultured pancreatic β-cells, thereby decreasing apoptotic rates in human endothelial cells and preventing the necrosis of murine calvaria pre-osteoblasts 33-35. Existing literature shows that the toxicity of PFCs is related to the carbon chain length 36. However, the molecular structure of PFTBA resembles a clover-leaf. We have previously demonstrated that the PFTBA hydrogel could protect Schwann cells in hypoxia and was safe in normoxic and hypoxic environments 22. Several PFC emulsions have been clinically tested as artificial blood substitutes with some success. Oxygent (Alliance Pharmaceutical Corp.), an emulsion containing perflubron, has shown the most promising results in terms of biocompatibility in Phase I and II clinical trials 37. In the present study, we also found that *in vitro* SC survival was comparable across the normoxia groups with or without PFTBA. Overall, toxicity data on these chemicals are still insufficient to assess safety in humans, and further studies are needed to examine this issue.

In tissue engineering, oxygen delivery methods are essential for tissue survival and function. Various methods have been investigated to deliver oxygen to scaffolds, such as hemoglobin-based oxygen carriers (HbOCs), peroxide-based oxygen generators, and photosynthetic biomaterials. HbOCs work similar to the hemoglobin in red blood cells; oxidation of hemoglobin to methemoglobin, which releases reactive ferryl ions and radicals, was the primary cause for oxidative stress and tissue toxicity observed with clinically-tested HbOCs 38. PFTBA-based biomaterials could be advantageous in providing oxygen to cells directly without any chemical reaction. Moreover, PFTBA has fewer biocompatibility concerns than peroxides, which produce ROS as a byproduct of its chemical reactions 39, 40. The photosynthetic biomaterials could provide tunable oxygen delivery without compromising the mechanical integrity of tissue constructs 41. However, it could only deliver oxygen when exposed to light, limiting their application to the skin or some subcutaneous tissues. In addition, the duration of oxygen release is the main limitation of an oxygen carrier. In our previous study, we found that PFTBA-hydrogel could release oxygen only for 48 h. In the present study, PFTBA was encapsulated in core-shell fibers from which the oxygen was released up to 144 h. Thus, the duration of oxygen release was closely linked to the property of loading materials. PFTBA can be easily incorporated into biomaterials and prolong the time course of oxygen release. It was believed that the new biomaterials or structures could overcome the limitation of oxygen carriers, which held therapeutic significance in regeneration of aerobic tissue injuries.

In most tissues, a venous oxygen concentration < 6% creates mild hypoxic effects, whereas maximum hypoxic responses occur around 0.5-1% of oxygen 42. However, it is important to note that the exact definition of oxygen hypoxia varies between organs and is different between *in vitro* and *in vivo* conditions 43. In addition, due to the difficulties of measuring the exact tissue oxygen levels experimentally, as well as the utilization kinetics of oxygen varies in the different tissue, it is still challenge to clarify the release kinetics of the oxygen that is fit into different models 44. In the present study, we provide the release kinetics of oxygen from PFTBA-based materials, showing beneficial effects on peripheral nerve regeneration. However, further studies to examine the release kinetics of oxygen in other models are still needed.

Over the last decade, electrospinning has emerged as a novel technique in regenerative medicine 45, 46. The application of an electrical field in the collector and spinneret through high voltages can enhance the solidification of polymers, increasing their size in the absence of post-treatment processing 47, 48. PCL is an aliphatic polyester that degrades slowly by either chemical hydrolysis of its ester bonds to caproic acid and its oligomers or through enzymatic hydrolysis 49. The sound elongation properties, excellent biocompatibility, and ease of electrospinning have made PCL useful for a coaxial electrospinning application. In the present study, we coated PCL membranes with poly-l-lysine (PLL), which is commonly used as a coating to enhance cell adhesion in standard cell culture processes. It can also promote cell spreading, proliferation, and differentiation on a hydrophobic surface 50. The coaxial electrospinning was explored to encapsulate water-soluble bioactive agents into core-shell structures, permitting the addition of bioactive agents into the fibers 45, 51. We reasoned that the core-shell structures could act as reservoirs for oxygen delivery using PFTBA, allowing the controlled release of oxygen through the fiber shell or fiber end in hypoxic conditions, enhancing the survival of SCs, and promoting nerve recovery and functionality., We found that fine core-shell fiber structure could be achieved by adjusting parameters (chitosan concentration and chitosan/PCL ratio)with only 5 sets of parameters in the electrospinning process forming the core-shell fiber structure. With the increasing concentration of chitosan in the core, the diameter of fibers increased and clustered together. These findings suggested that the concentration of inner solution and spinnability of chitosan are key factors in core-shell structure formation. We selected 10% chitosan (chitosan: PCL, 1:6) to prepare the core-shell fibers, a parameter that achieved long duration of oxygen release. The prolonged release of oxygen from the fibers was probably attributable to the uniformly ultrafine structures and surface area for gas diffusion. The exact underlying mechanism, however, still requires further exploration.

Anatomically, the rat's sciatic nerve divides into two nerves (the tibial and the common peroneal nerve) in the back of the knee with a diameter smaller than ~0.94 mm. We noticed the longest distance between sciatic nerve outlet to branches was ~19 mm (Male SD rats, 230-250 g; Figure 5). Thus, we prepared the conduits with the 19 mm length. Furthermore, changes in the conduit inner diameter could alter growth factors' concentration and change mechanical support for growing axons. It has been reported that reconstruction with collagen conduits with an inner diameter of 1.5 mm generates a better nerve motor recovery than the 2.0 mm diameter conduits in a rat sciatic nerve defect model 52. Two reasons could explain this observation: (1) a decrease in conduit dead space could increase the concentration of growth factors released from the nerve stumps improving the quality of regenerating axons; (2) better mechanical support for growing axons with better size-matching nerve conduits could improve axonal direction and sustainability. Thus, we fabricated the conduits with inner diameter of 1.5mm in the present study.

We observed a loss of SC viability in fibers lacking PFTBA after 144 h of hypoxic conditions, confirming the damaging effects of oxygen deprivation. Using SEM, we also detected SC apoptosis when cells were cultured in hypoxic conditions, further confirming the damaging effects of hypoxia on SCs. Culturing SCs on electrospun fibers possessing PFTBA partially reversed the effects of hypoxia on survival and viability. Also, the morphology of the cells improved as assessed by SEM after 144 h of hypoxic conditions. These findings suggested that PFTBA-encapsulated fibers improve the survival of SCs for 144 h, consistent with similar studies performed in both SCs and MSCs 20, 22. To further develop PFTBA-based SC culture systems *in vitro*, we cultured SCs in 3D systems with or without PFTBA, and assessed their survival characteristics. We observed a higher percentage of live cells in 3D systems that contained both PFTBA gels and fibers, highlighting their ability to provide sustained oxygen release to SCs under hypoxic conditions. Furthermore, PFTBA in the core-shell fibers and hydrogels significantly increased NGF 53, BDNF 54, and VEGF 55 expression, all of which are growth factors that promote nerve regeneration during different recovery stages. SCs were able to secrete neurotrophins and produce extracellular matrix molecules to facilitate axonal outgrowth and elongation. However, we have to realize that the harvest of SCs might limit the SCs therapy from basic to clinical research, which still needs more studies to establish ideal sources of SCs in the future. These findings demonstrated the beneficial effects of PFTBA on cell survival and functionality in 2D and 3D *in vitro* systems, underscoring their utility to enhance the survival of SCs to promote nerve regeneration *in vivo*.

PFTBA increased SC survival in the nerve conduits* in vivo*, as evidenced by the higher number of GFP-SCs in the PFTBA fiber conduit + PFTBA/SCs/gel group at 7 and 14 days after implantation and the SCs were viable after 14 days due to the higher level of revascularization 56. These findings demonstrated that PFTBA could increase SC survival for at least 14 days in repairing nerve defects, contributing to axonal regeneration.

In nerve injury repair, the reconnection of peripheral nerves is a complicated process 57. Here, we observed that regenerated nerves had higher levels of remyelinated axons that were evenly distributed across the nerve conduit in the PFTBA fibers and PFTBA/SCs/gels, providing morphological evidence for successful regeneration of a large number of nerve fibers in the scaffolds. We also performed electrophysiological analysis and retrograde labeling to evaluate functional recovery and axonal transport 58 and observed greater numbers of FG-positive neurons, larger CMAPs, and more rapid NCVs with PFTBA fiber conduit + PFTBA/SCs/gel, indicating that the PFTBA-based systems may promote the nerve reinnervation of target muscles 59, 60. In this context, the walking ability, due to the requirement of controlled sensory inputs, cortical integration and motor responses, has been widely studied for assessing restorative capacity during sciatic nerve repair 61. In our study, SFI was introduced to evaluate walking ability and quantify functional recovery of regenerating axons. Consistent with the findings of morphometric and electrophysiological analyses, improved motor functional recovery was achieved with PFTBA fiber conduit + PFTBA/SCs/gel. These findings indicated that a more efficient oxygen delivery by PFTBA-based systems could enhance axonal regeneration, leading to improved neurologic functional recovery.

It has been reported that engineered tissues larger than 1 cm^3^ are generally hypoxic in their central regions 62. Microvessels for oxygen transportation in regenerating tissues have been shown to infiltrate at ~2 mm depths after 14 days of graft implantation 33. Providing oxygen is therefore challenging during tissue regeneration, especially during the early stages of tissue repair. It has been reported that formation of microvessels in regenerated tissues requires at least 2 weeks during which oxygenation is critical before regenerated microvessels take over 63 Thus, we defined the early stages as a period before formation of microvessels (≤ 2 weeks). The growth of newly formed blood capillaries peaks at ~600 μm/day 64. The optimal size of the PFTBA system supporting the viability of seeded-cells during blood vessel ingrowth was calculated as ~7.2 mm (~600 μm/day × 6 days × 2 directions = ~7,200 μm). By this concept, cell scaffolds containing PFTBA showed favorable therapeutic potential for treating human peripheral nerve injuries. PFTBA was capable of providing oxygen to local cells and enhancing survival of cells for 14 days. We previously reported that PFTBA could provide quick and limited oxygen to the injury site beneficial for vascularization. We also demonstrated that PFTBA could improve the survival of SCs by promoting VEGF expression and enhancing vascularization 5. However, the quick release kinetics of PFTBA could not reverse severe hypoxia and limited its application in the later stage of nerve repair. It was, therefore, necessary to enhance nerve regeneration by coupling PFTBA's oxygen supply in the early stage with the late stage vascularization.

In this study, by using the coaxial electrospinning technique, we fabricated the PFTBA oxygen-carrying system to provide oxygen to cells within the tissue engineering scaffold to promote their survival until the formation of vascular networks. This strategy would open up new research avenues for numerous applications in regenerative engineering, such as wound healing, bone defect repair, cardiac repair, liver transplantation, and central nervous system regeneration. By providing a novel strategy to supply oxygen for extended periods prior to vascularization during tissue defect repairs, PFTBA-based oxygen carriers are might be promising tools in the 3D bioprinting field for manufacturing human tissues and organs.

## Summary

PFTBA systems can provide oxygen for SCs and promote their survival in hypoxic conditions. In this study, the PFTBA-based oxygen carrier system was prepared by coaxial electrospinning to prolong the time course of oxygen release. Core-shell structures were fabricated and optimized and the oxygen kinetics of PFTBA-encapsulated core-shell fibers was evaluated. *In vitro* studies showed that, under hypoxic conditions, the PFTBA core-shell fibers were capable of providing oxygen to SCs for extended periods, resulting in increased survival and upregulated NGF, BDNF, and VEGF expression in 2D and 3D culture systems. *In vivo* analysis revealed that the majority of SCs in the PFTBA conduit remained viable 14 days post-implantation. The axons treated with PFTBA oxygen carrier scaffolds showed improved axonal regeneration, remyelination, and recovery. In conclusion, the PFTBA system improved axonal regeneration and neurological functionality and recovery of nerve defects. Collectively, our data illustrated the potential of PFTBA as an oxygen carrier to overcome the hypoxic environment encountered during nerve injury.

## Supplementary Material

Supplementary figure, tables, and movie legends.

Supplementary movie 1.

Supplementary movie 2.

Supplementary movie 3.

Supplementary movie 4.

## Abbreviations

FG: Fluoro-Gold
FITC: fluorescein isothiocyanate
IHC: Immunohistochemistry
PFA: paraformaldehyde
PCL: polycaprolactone
PFCs: Perfluorocarbons
PFTBA: perfluorotributylamine
PLL: poly-l-lysine
PNI: peripheral nerve injury
SCs: Schwann cells
SD: Sprague Dawley
SEM: scanning electron microscope
SFI: sciatic function index
TEM: transmission electron microscope
