# Oxygen carrier in core-shell fibers synthesized by coaxial electrospinning enhances Schwann cell survival and nerve regeneration: Erratum

**DOI:** 10.7150/thno.113747

**Published:** 2025-05-15

**Authors:** Teng Ma, Yafeng Yang, Xin Quan, Lei Lu, Bing Xia, Jianbo Gao, Fengyu Qi, Shengyou Li, Laihe Zhao, Liangwei Mei, Yi Zheng, Yanbing Shen, Zhuojing Luo, Yan Jin, Jinghui Huang

**Affiliations:** 1 Institute of Orthopaedics, Xijing Hospital, Fourth Military Medical University, Xi'an 710032, China.; 2 Research and Development Center for Tissue Engineering, School of Stomatology, Fourth Military Medical University, Xi'an 710032, China.; 3 Hospital of 76th Group Army of PLA, Xining, 810000, China.; 4 Department of Orthopedics, Fourth Medical Center of Chinese PLA General Hospital, Beijing, 100048, China.; 5 Department of Plastic Surgery, Xijing Hospital, Fourth Military Medical University, Xi'an 710032, China.; 6 Department of Oral Anatomy and Physiology, State Key Laboratory of Military Stomatology, School of Stomatology, Fourth Military Medical University, Xi'an, 710032, China.; 7 Department of Orthopedics, General Hospital of Central Theater Command of PLA, Wuhan, 430070, China.

The authors regret that the original version of our paper, unfortunately, contained an incorrect picture in Figure 3A, where the images for the Fibers+PFTBA-gel group were mistakenly used for the PFTBA Fibers+gel group. The correct version of Figure 3A is shown below.

The correction made in this erratum does not affect the original data and conclusions. The authors apologize for any inconvenience that the errors may have caused.

## Figures and Tables

**Figure 3:**
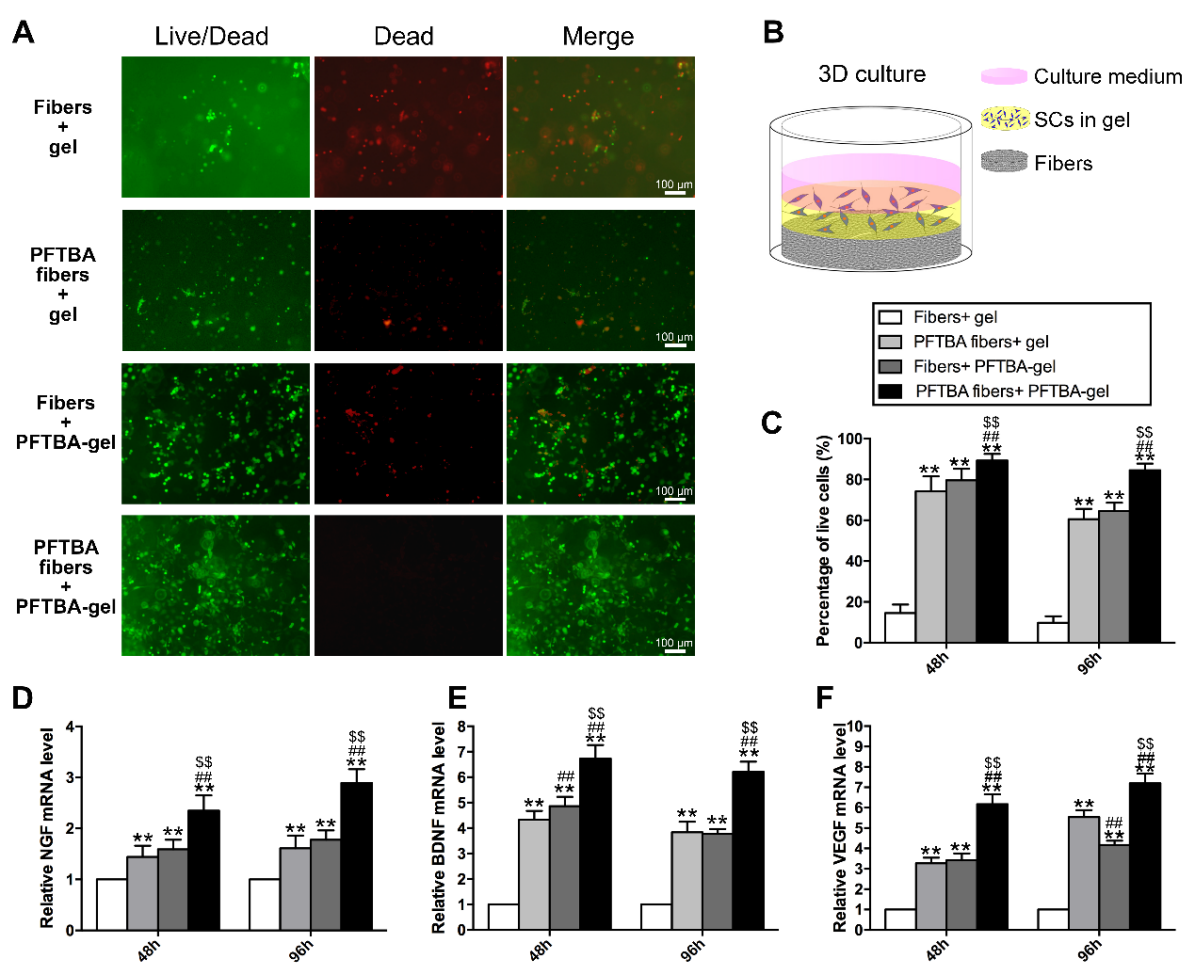
Representative images of Live-Dead staining of the 3D cultured Schwann cells in each group (A). Schematic diagram of the process of preparing the 3D culture matrix (B). Percentages of living SCs for each group are shown in (C). mRNA levels of NGF (D), BDNF (E), and VEGF (F) in each group at 48 and 96 h after hypoxia.

