# Supplementary material for: Oxygen carrier in core-shell fibers synthesized by coaxial electrospinning enhances Schwann cell survival and nerve regeneration
Source: Theranostics. 2020 Jul 11;10(20):8957–73. doi: 10.7150/thno.45035 (PMC7415813; doi:10.7150/thno.45035)
Supplement: Supplementary file 1 — Supplementary figure, tables, and movie legends. [file thnov10p8957s1.pdf]

Supplementary Material

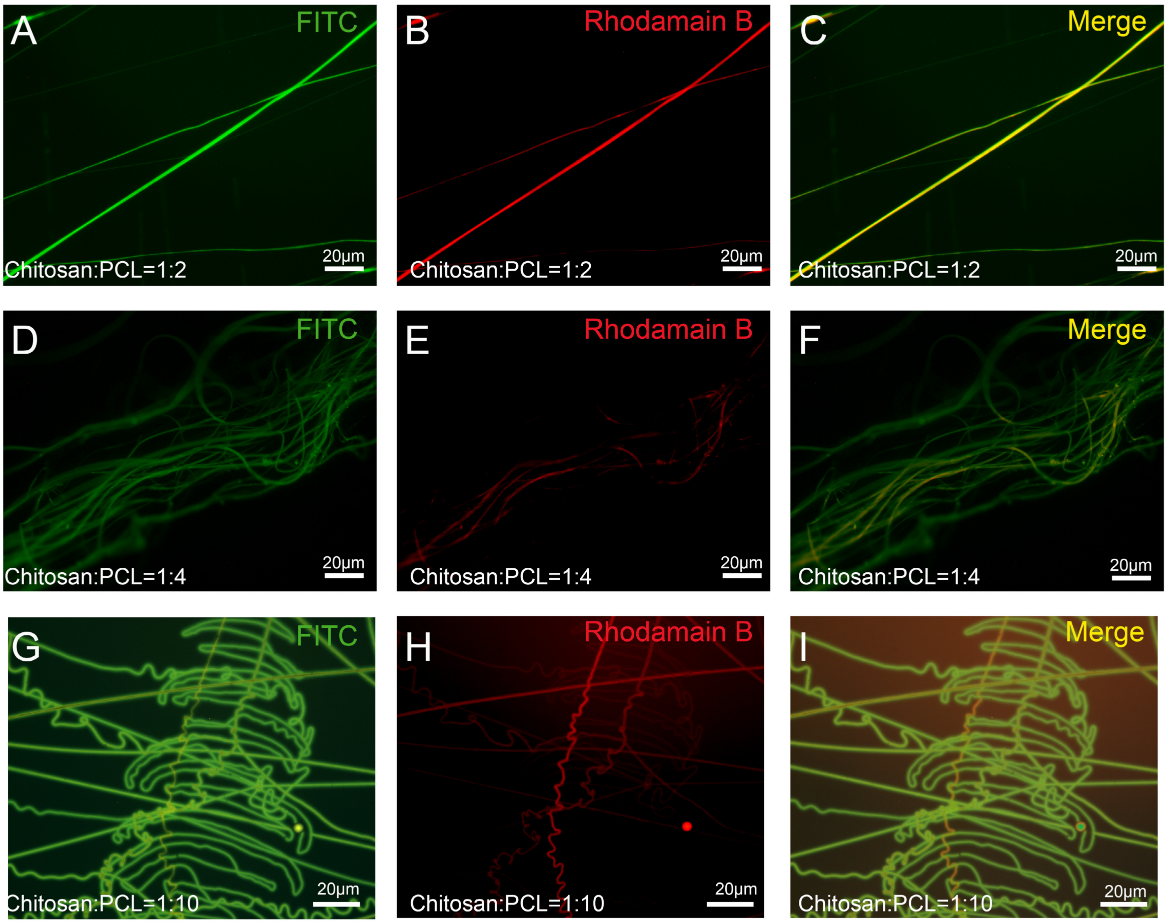

Figure S1. The microstructural appearance of core-shell fibers under fluorescence microscopy. Ratio between the core solution and shell solution was 1:2 (A-C), 1:4(D-F) and 1:10 (G-I). To observe of the electrospinning process, Rhodamine B (Red) was mixed into the core solution and fluorescein isothiocyanate (FITC, green) was added to the shell solution.

Gene	GenBank Accession no.	Direction	Sequence	Length (bp)
Ngf	NM_001277055.1	Upper	5’ATCGCTCTCCTTCACAGAGTTT3’	217
		Lower	5’ TGTACGGTTCTGCCTGTACG3’	
Bdnf	NM_001270630.1	Upper	5’ GTCGCACGGTCCCCATTG 3’	246
		Lower	5’ ACCTGGTGGAACTCAGGGT3’	
Vegfa	NM_031836.3	Upper	5'CGGTTCCAGAAGGGAGAGGA3'	237
		Lower	5' ACTTCACCACTTCATGGGCT 3'	
Actb	NM_031144.3	Upper	5’GCAGGAGTACGATGAGTCCG 3’	74
		Lower	5’ ACGCAGCTCAGTAACAGTCC 3’	

Table S1. Primer sequences used for the real-time PCR

Table S2. Number of rats per group and time point allocated to different assessments.

	Autograft	Fibers conduit + SCs/gel	PFTBA fibers conduit+ SCs/gel	Fibers conduit+ PFTBA/SCs/gel	PFTBA fibers conduit+ PFTBA/SCs/gel
**7 days**					
The analysis of cell survival after surgery		6	6	6	6
**14 days**					
The analysis of cell survival after surgery		6	6	6	6
**6 weeks**					
Axonal regeneration and functional recovery assessment△	6	6	6	6	6
Fluoro-Gold retrograde tracing assessment	6	6	6	6	6
Immunohistochemistry assessment	6	6	6	6	6
**12 weeks**					
Axonal regeneration and functional recovery assessment△	6	6	6	6	6
Fluoro-Gold retrograde tracing assessment	6	6	6	6	6
Total number	30	42	42	42	42

△ Axonal regeneration and functional recovery assessment containing morphometric analysis of sciatic nerve,

behavioral analysis, electrophysiological assessment and histological analysis of target muscle

Movie legends

Movie S1. Processes of fabrication of core-shell fibers by coaxial electrospinning. Ratio between the core solution and shell solution was 1:6.

Movie S2. Processes of fabrication of core-shell fibers by coaxial electrospinning. Ratio between the core solution and shell solution was 1:4.

Movie S3. Processes of fabrication of core-shell fibers by coaxial electrospinning. Ratio between the core solution and shell solution was 1:2.

Movie S4. Processes of fabrication of core-shell fibers by coaxial electrospinning. Ratio between the core solution and shell solution was 1:10.
